# Research Progress in the Application of Nanotechnology in Fracturing: A Review

**DOI:** 10.3390/nano15201539

**Published:** 2025-10-10

**Authors:** Lei Liang, Huiru Lei, Qinwen Zhang, Wei Zhao, Dong Liao, Dong Wang, Yujia Xiong, Lang Liu, Hualin Liu, Zilai Mei

**Affiliations:** CCDC Geological Exploration and Development Research Institute, Chengdu 610051, China

**Keywords:** nanotechnology, hydraulic fracturing, fracturing fluid, proppant, unconventional reservoirs, interface regulation

## Abstract

Hydraulic fracturing is a core stimulation technology for enhancing hydrocarbon production. However, it faces significant technical bottlenecks in unconventional reservoirs. These bottlenecks include poor adaptability to high-temperature and high-salinity environments, water-sensitive formation damage, and insufficient long-term fracture conductivity. Nanotechnology leverages unique properties of nanomaterials, such as surface effects, quantum size effects, and designability. Nanotechnology offers systematic solutions for optimizing fracturing fluids, enhancing proppant performance, and innovating waterless fracturing techniques. This review outlines the current status of fracturing technology, exploring the role of nanoparticles in improving fluid rheology, proppant strength, and interface regulation, and discusses future challenges. Studies show that nanomodified fracturing fluids can increase high-temperature viscosity retention by over 300%. Meanwhile, waterless fracturing reduces water consumption by 80%. Despite challenges in particle agglomeration and cost, nanotechnology demonstrates significant potential in boosting recovery and reducing environmental impact. Nanotechnology is positioned as a transformative technology for future unconventional resource development.

## 1. Introduction

The global demand for energy continues to rise. Unconventional hydrocarbons, including shale oil, tight gas, and coalbed methane, have emerged as critical components of the energy mix [1]. However, these resources are characterized by ultra-low permeability and complex geological conditions. This makes their extraction heavily reliant on hydraulic fracturing. As a core stimulation technique, hydraulic fracturing creates artificial fractures. These fractures connect hydrocarbon-bearing zones to the wellbore, enabling viable production [2,3].

The development of unconventional reservoirs has exposed inherent limitations in traditional fracturing technologies. These technologies may not adequately address the unique challenges posed by these formations [4,5]. High-temperature and high-salinity environments are prevalent in deep unconventional reservoirs. They destabilize conventional fracturing fluids, leading to viscosity loss and reduced fracture propagation efficiency [6,7,8,9]. Water-sensitive formations are rich in clay minerals. When exposed to water-based fluids, they suffer severe permeability damage. This is because clay hydration swells pore throats and blocks flow pathways [10,11].

Additionally, proppants are essential for maintaining fracture conductivity. But they often fail under the high closure stresses of deep reservoirs. At the same time, traditional fracturing involves large water consumption and environmental risks. These factors further complicate sustainable development [12,13].

These challenges not only hinder production efficiency but also raise concerns about resource waste and ecological impact. This is particularly true in regions facing water scarcity or strict environmental regulations [14]. Nanotechnology has the ability to manipulate matter at the nanoscale, making it a promising solution. By harnessing the unique physical and chemical properties of nanomaterials—such as enhanced surface reactivity, tunable functionality, and size-dependent effects—researchers have developed innovative approaches. These approaches optimize fracturing fluids, strengthen proppants, and even enable waterless fracturing [15].

This review synthesizes the latest advancements in nanotechnology applications for fracturing. It analyzes their mechanisms of action and discusses the path forward for overcoming remaining obstacles. It also highlights the technology’s potential to redefine unconventional resource development [16,17,18].

## 2. Challenges of Fracturing Technology

### 2.1. Unconventional Reservoir Development

Unconventional reservoirs are defined by ultra-low permeability (less than 0.1 mD) and high clay content. They represent a growing share of global hydrocarbon production, but their development is inherently challenging. Unlike conventional reservoirs, which allow fluid flow through natural pathways, unconventional reservoirs require hydraulic fracturing. This process creates and maintains artificial flow channels. This dependency amplifies the impact of any inefficiencies in fracturing techniques.

Traditional water-based fracturing fluids are cost-effective, but they exacerbate formation damage in water-sensitive reservoirs. Clay minerals, such as montmorillonite, readily hydrate upon contact with water. This hydration causes swelling that blocks pore throats and reduces permeability by 40–60% [19]. For instance, sodium-based bentonite expands by 65% in conventional fluids. This expansion effectively plugs microchannels that are critical for hydrocarbon flow. Such swelling not only reduces initial productivity but also complicates long-term reservoir management. Reverse damage is often irreversible in these cases.

Polymer-based crosslinked fluids are designed to enhance viscosity through borate or zirconium ion crosslinking. However, they introduce a different set of issues. Their residual polymers—typically 6–10% of the original volume—form filter cakes on fracture surfaces. These filter cakes reduce conductivity by over 50% [20]. This residual material acts as a physical barrier. It limits hydrocarbon migration even after fractures are created. Oil-based fluids avoid water sensitivity, but they are economically prohibitive. Their cost is 4–6 times that of water-based fluids. Moreover, they pose significant environmental risks, including challenges in toxic waste disposal [21]. These factors make them unsuitable for large-scale applications.

Proppant performance is another critical bottleneck. This is especially true in deep reservoirs where closure stresses exceed 69 MPa. Quartz sand is the most common proppant in shallow layers (less than 2000 m), but the grains may fracture under high stress. Once fractured, it loses the ability to prop open fractures. Ceramic proppants are stronger, but they have a high density of 3.8 g/cm^3^. This high density increases pumping pressure by 25% and leads to significant conductivity loss—30% over 180 days. This trade-off between strength and density of proppants limits their effectiveness in deep, high-stress environments.

**Figure 1:**
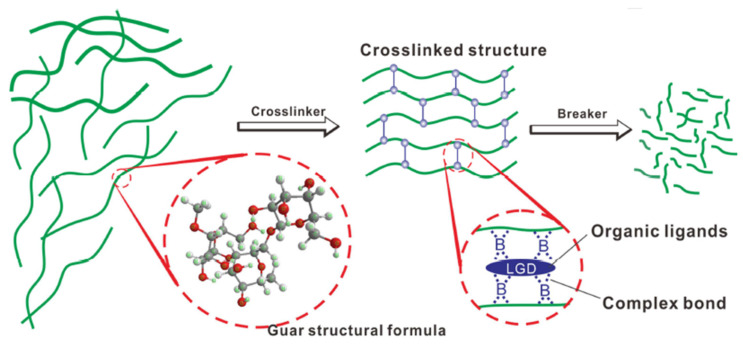
Crosslinking and breaking process of the guar gel. Reproduced with permission from [22]. Copyright Elsevier, 2019.

Guar gel is a widely used polymer matrix in conventional fracturing fluids, and its crosslinking with agents like borate directly determines fluid viscosity and fracturing efficiency. The crosslinking and breaking process of guar gel [22] is specifically illustrated in Figure 1.

### 2.2. Conventional Fracturing Fluids

Conventional fracturing fluids have multiple, often conflicting, roles. They need to generate sufficient viscosity to transport proppants, minimize fluid loss into the formation, and break down easily after fracturing to avoid residual damage. Balancing these functions has proven elusive. This has led to performance gaps that hinder efficiency.

Rheological instability is a primary concern. Viscoelastic surfactant (VES) fluids rely on micellar networks to achieve viscosity. But they degrade rapidly at high temperatures. Above 93 °C (200 °F), micelles disintegrate, causing viscosity to drop by 70% [23]. For example, at 104 °C (220 °F), VES fluid viscosity plummets from 150 mPa·s to 40 mPa·s. This rapid drop accelerates proppant settlement and reduces the effectiveness of fracture propagation. This temperature sensitivity limits their use in deep, high-temperature reservoirs. These reservoirs are increasingly targeted for their resource potential.

Inadequate filtrate control further compounds issues. Microscale additives like calcium carbonate fail to seal the nanoscale pores of shale reservoirs. These pores typically range from 10 nm to 100 nm. This failure results in fluid loss of 30–40% [24]. This loss not only wastes expensive fracturing fluids but also induces water blockage. Water blockage is a phenomenon where trapped water reduces relative permeability to hydrocarbons. It lowers gas well productivity by 20–30%. The cumulative effect of fluid loss and water blockage can render even successful fractures economically unviable.

**Figure 2:**
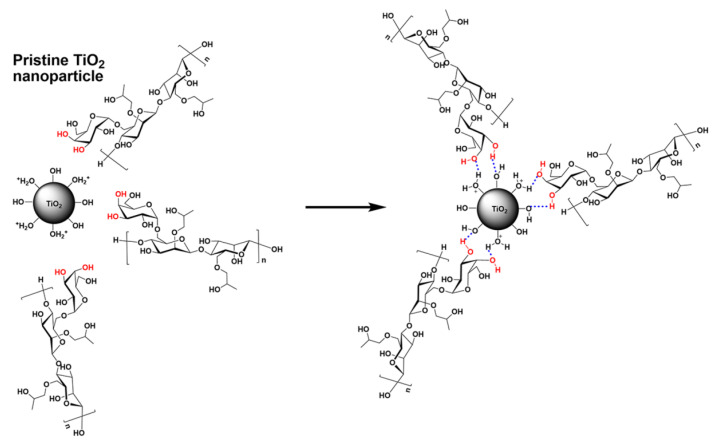
Cross-linking mechanism between TiO_2_ and hydroxypropyl guar (HPG). Reproduced with permission from [25]. Copyright American Chemical Society, 2015.

Post-fracturing damage from residual polymers exacerbates these challenges. After breaking, polymer-based fluids leave behind 30–40% residual oligomers with a molecular weight greater than 5000. These oligomers persist in pore throats and limit conductivity recovery to 60–70%. TiO_2_ nanoparticles can form stable cross-linking structures with hydroxypropyl guar (HPG) to enhance fracturing fluid stability, and the specific cross-linking mechanism [25] is shown in Figure 2.

This residual material is particularly problematic in ultra-low permeability reservoirs. Even minor blockages in these reservoirs can drastically reduce flow rates. Together, these issues highlight the need for innovative solutions. These solutions should address the multifunctional demands of fracturing fluids without compromising performance.

## 3. Nanotechnology Innovations in Fracturing

### 3.1. Unique Properties

Nanoparticles, which range from 1 nm to 100 nm in size, possess distinct properties. These properties make them ideal for addressing fracturing challenges. They stem from the nanoparticles’ small size and high surface-to-volume ratio. These properties enable interactions with fluids, proppants, and reservoir rocks at the molecular level. This offers precise control over material behavior.

The surface effect is a hallmark of nanomaterials. It arises from the high proportion of surface atoms relative to bulk atoms. For example, SiO_2_ nanoparticles exhibit a specific surface area of 300 m^2^/g. This creates abundant active sites for interactions with polymers. These interactions include hydrogen bonding (with an energy range of 20–40 kJ/mol) and electrostatic forces. They strengthen crosslinked networks in fracturing fluids, enhancing stability under harsh conditions [26]. This ability to reinforce molecular structures addresses a key limitation of conventional fluids. Conventional fluids rely on weaker intermolecular forces that degrade at high temperatures or under stress.

Quantum size effects are observed in nanoparticles like TiO_2_, which has a 3.2 eV bandgap. These effects introduce novel functionalities such as photocatalysis. TiO_2_ nanoparticles can degrade residual polymers under UV exposure. This enables a “self-cleaning” mechanism that reduces post-fracturing damage [27]. This property directly targets the problem of polymer residue. It offers a passive solution to improve conductivity recovery.

Dielectric confinement effects are exemplified by multi-walled carbon nanotubes (MWCNTs), which exhibit a dielectric constant of 1000 under specific preparation conditions (e.g., high purity and controlled aspect ratio) [28]. This functionality is critical for optimizing fracturing operations. Real-time monitoring of fracture networks remains a challenge in conventional techniques.

### 3.2. Preparation and Dispersion

The effectiveness of nanomaterials in fracturing depends on precise synthesis and dispersion. Agglomeration can negate their nanoscale advantages. Advanced preparation methods have been developed to produce uniform, stable nanoparticles. These nanoparticles are tailored for fracturing applications. Inert gas condensation produces metal oxide nanoparticles, such as TiO_2_ and ZnO. These nanoparticles have controlled sizes (ranging from 10 nm to 50 nm) and high purity (greater than 99%). This method ensures consistent particle properties, which is essential for reproducible performance.

For example, TiO_2_ nanoparticles synthesized this way form stable hydrogen-bonded networks with hydroxypropyl guar at a pH range of 2–4. They maintain functionality up to 160 °C [29]. Such stability is critical for applications in high-temperature reservoirs. Material consistency directly impacts performance in these reservoirs. Solid-state milling with surface modification addresses agglomeration. Agglomeration is a common issue in high-salinity environments. For instance, silane-coupled SiO_2_ nanoparticles treated with KH550 exhibit a zeta potential shift. The shift goes from −15 mV to +25 mV in 3.5% NaCl solution. This shift enhances electrostatic repulsion, reducing agglomeration by 60% [30]. By improving dispersion, this technique ensures nanoparticles remain active in brine-rich formations. Conventional additives often fail in these formations.

Microemulsion synthesis enables the production of core–shell nanoparticles, such as Fe_3_O_4_@SiO_2_. These nanoparticles combine functionality with controlled release. The SiO_2_ shell of these nanoparticles dissolves in response to pH changes. This dissolution releases breaker agents and reduces gel breaking time by 50% [31]. This targeted release mechanism optimizes fluid breakdown. It minimizes residual damage while ensuring timely fracture conductivity restoration.

## 4. Applications

### 4.1. Nanomodified Fracturing Fluids

#### 4.1.1. High-Temperature Resistant Crosslinking Systems

Nanoparticles have revolutionized the design of high-temperature fracturing fluids. They reinforce crosslinked networks to achieve this. TiO_2_ nanoparticles, with sizes ranging from 6 nm to 14 nm, form hydrogen-bonded 3D structures with guar polymers. These structures sustain 480 mPa·s viscosity at 150 °C under 170 s^−1^ shear. This viscosity is 3.2 times higher than that of conventional borate systems [32]. This stability arises from the dual role of TiO_2_: it acts as a crosslinking agent and reinforces the polymer matrix against thermal degradation.

SiO_2_ nanoparticles functionalized with boronic acid (-NH_2_ groups) further enhance performance. They form covalent N-B bonds with guar to do this. This reduces borate usage by 70% while maintaining 85% viscosity retention under 100 MPa stress [33]. The covalent bonds resist dissociation at high temperatures. This addresses the failure mechanism of traditional borate crosslinkers, which rely on reversible ionic interactions. Together, these advancements enable fracturing in deep, high-temperature reservoirs. These reservoirs were previously deemed inaccessible with conventional fluids.

#### 4.1.2. Filtrate Control and Clay Stabilization

For nanoscale pores, 20 nm SiO_2_ nanoparticles form a dense filter cake. They use a “bridging-filling” mechanism to achieve this [34]. At 85 °C and a pressure difference of 100 psi, the filtration loss of the fracturing fluid is 1.2 mL/min. This fluid contains 1% SiO_2_ nanoparticles.

In comparison, the traditional sand particle system has a filtration loss of 1.8 mL/min. This represents a 33% reduction for the nanoparticle-containing fluid. When the particle size of the nanoparticles is 1/3–1/2 of the pore size, the nanoparticles are first adsorbed at the entrance of the pore throat. This forms an initial plugging layer.

Subsequent nanoparticles stack via van der Waals forces. Finally, the permeability of the filter cake decreases to 5 × 10^−3^ μm^2^. This is only 1/5 of the permeability of the traditional filter cake. The surface hydroxyl density of hydrophilic nano-Al_2_O_3_ is 1.5 OH/nm^2^. It can inhibit clay swelling through electrostatic repulsion in water-sensitive formations [35]. Scanning electron microscopy (SEM) observations show results after treatment with nano-Al_2_O_3_.

The interlayer spacing of sodium-based bentonite decreases from 1.5 nm to 1.2 nm. The swelling rate reduces from 55% to 30%. X-ray energy dispersive spectroscopy (EDS) analysis reveals additional details. The nanoparticles form a protective film on the clay surface. This film blocks the direct contact between water molecules and montmorillonite crystal layers. Meanwhile, the zeta potential is increased to +35 mV. This enhances the repulsive force between particles.

#### 4.1.3. Smart Gel-Breaking and Rheology Modification

Nanotechnology enables precise control over gel breaking and fluid rheology. This optimization improves post-fracturing cleanup and proppant transport. pH-responsive Fe_3_O_4_@SiO_2_ nanocapsules, with a size of 50 nm, reduce gel breaking time. The time goes from 60 min to 25 min in acidic formations. Additionally, the polymer residue is less than 5% [36]. The pH-sensitive shell dissolves selectively in low-pH environments. It releases breakers where and when needed, avoiding premature degradation in the wellbore.

**Figure 3:**
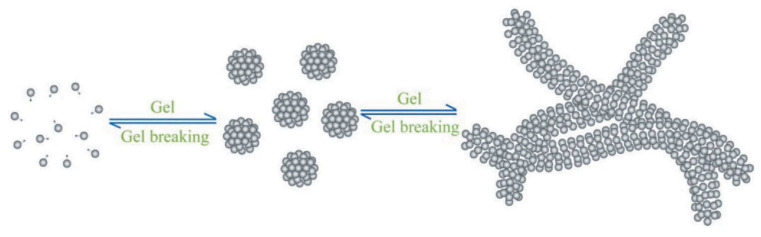
Mechanism of VES as fracturing fluid thickener. Reproduced with permission from [37]. Copyright Taylor & Francis Group, 2018.

MgO nanoparticles, used at a 0.5 wt% concentration, enhance the elasticity of VES fluids. They increase the storage modulus (G′) from 12 Pa to 60 Pa. This “strong elastic” behavior slows proppant settling. The settling rate decreases from 10 cm/min to 4 cm/min. This enables transport of large proppants (40/70 mesh). Viscoelastic surfactant (VES) fluids rely on micellar networks to achieve viscosity, but they degrade rapidly at high temperatures; the mechanism of VES [37] acting as a fracturing fluid thickener is detailed in Figure 3. The nanoparticles interact with surfactant micelles. They stabilize the micelle structure and prevent disintegration at high temperatures. This addresses a key limitation of VES fluids in deep reservoirs.

### 4.2. Nanoproppants for Enhanced Performance

#### 4.2.1. High-Strength Composite Proppants

Nanomodification improves proppant strength and durability. This is critical for deep reservoirs with high closure stresses. CNT-modified bauxite proppants increase compressive strength by 25%. The strength goes from 80 MPa to 100 MPa. They also maintain 85% conductivity under 70 MPa stress. This conductivity is 55% higher than that of conventional ceramic proppants [38]. CNTs act as nanoscale “reinforcing bars” at grain boundaries. They inhibit crack propagation and distribute stress evenly across the proppant matrix. This structure addresses the trade-off between strength and weight. CNTs add minimal density while significantly enhancing mechanical performance.

Silica-coated proppants reduce CO_2_-induced dissolution by 40%. They maintain conductivity in acidic reservoirs with a pH of 3.5 [39]. The silica layer acts as a chemical barrier. It prevents reaction between proppant minerals and acidic fluids. Reaction between proppant minerals and acidic fluids is a common issue in reservoirs with high CO_2_ content. This coating extends proppant lifespan. It ensures long-term fracture conductivity.

#### 4.2.2. Wettability Modification and Conductivity Optimization

Nanocoatings alter fracture surface wettability. This modification enhances hydrocarbon flow. Superhydrophobic SiO_2_ coatings have a 135° contact angle. They reduce the water relative permeability by 35% while increasing oil flow by 25% [40]. By lowering surface energy from 75 mN/m to 25 mN/m, these coatings repel water. They also expand effective pore throats, which go from 5 μm to 8 μm. This expansion facilitates oil migration.

Cationic Al_2_O_3_ nanoparticles have a −40 mV charge. They form double electric layers with anionic surfactants. This reduces capillary pressure by 30% and mitigates water blockage in tight sandstones [41]. This electrostatic interaction weakens the adhesion of water to rock surfaces. It allows hydrocarbons to displace trapped water more effectively. Together, these modifications address the long-standing challenge of water blockage. Water blockage is a major contributor to productivity loss in unconventional reservoirs.

### 4.3. Waterless Fracturing and Environmental Sustainability

#### 4.3.1. Nanoparticle-Stabilized Foam Systems

Nanoparticles enable waterless fracturing. This is useful in arid regions or water-sensitive reservoirs. They stabilize CO_2_ foams to achieve this. SiO_2_-stabilized CO_2_ foams exhibit an 8 h half-life. In comparison, surfactant-only foams have a 2 h half-life. They also maintain 120 cp viscosity at 95% quality. This reduces water usage by 80% in the Permian Basin [42]. Methyl-silanized nanoparticles enhance adsorption energy at the gas–liquid interface. The energy is −45 kJ/mol, compared to −20 kJ/mol for surfactants. This enhancement suppresses bubble coalescence and improves foam stability. This technology not only conserves water but also reduces the volume of flowback liquids. This lowers treatment costs and environmental risks.

#### 4.3.2. Liquid CO_2_/N_2_ Fracturing with Nanoparticle Additives

Liquid CO_2_ fracturing can be enhanced by 30 nm Al_2_O_3_ nanoparticles. It overcomes its low viscosity (2 mPa·s) to transport proppants to 300 m fractures. Al_2_O_3_ increases viscosity to 6 mPa·s. This slows proppant settling from 5 cm/min to 1 cm/min [43].

Thermal shock from CO_2_ vaporization (with a temperature change ΔT greater than 50 °C) induces additional microcracking. Meanwhile, 95% flowback efficiency eliminates liquid waste. This system aligns with environmental goals. CO_2_ can be sequestered post-fracturing, reducing greenhouse gas emissions.

### 4.4. Nanotechnology for Monitoring and Multifunctional Integration

Magnetic Fe_3_O_4_ nanoparticles, with a size of 50 nm, enable high-resolution NMR imaging of fracture networks. The resolution is 50 μm, which is 4 times more precise than fluorescent tracers [44]. In the Marcellus Shale, this technology identified undrained zones. It guided refracturing and boosted production by 10%. Graphene-polymer composites have a 260 m^2^/g surface area. They combine high viscosity (200 mPa·s at 120 °C) with 85% salt tolerance. This makes them ideal for high-salinity tight oil reservoirs [45]. These multifunctional materials integrate fluid stability, monitoring, and environmental compatibility. They address multiple challenges simultaneously.

### 4.5. Challenges and Future Directions

#### 4.5.1. Technical Hurdles

Agglomeration in high-salinity environments remains a critical issue. Ionic screening reduces zeta potential and increases agglomeration rates by 3 times. Block copolymer dispersants, such as PAA-PEO, are being developed. They maintain stability through steric hindrance [46]. Cost and scalability hinder widespread adoption. Nanoscale SiO_2_ costs 50/kg, which is 10 times the cost of traditional additives. Waste-derived sources, such as fly ash and coal gangue, could reduce costs. They may bring costs to less than 10/kg, making nanotechnology economically viable [47]. Environmental risks also exist. One such risk is potential microbial membrane penetration by sub-10 nm nanoparticles. Ecotoxicological assessments are required. These assessments ensure biodegradability and prevent long-term ecological impact [48].

#### 4.5.2. Future Research Priorities

Multiscale material design aims to integrate nanoparticles, micron fibers, and macro proppants. The target is 15% higher packing density and 25% improved conductivity [49]. Biodegradable nanoparticles, such as chitosan and starch-based ones, seek to achieve zero-residue fracturing. They decompose into water and CO_2_ [50].

**Figure 4:**
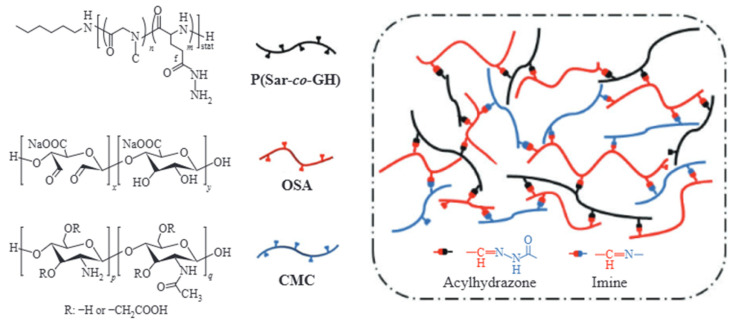
Preparation of dual dynamic covalent bond crosslinked hydrogels [51].

Intelligent responsive systems are another focus. For example, temperature/pH dual-responsive PNIPAM-modified SiO_2_ will enable adaptive fracture sealing. This sealing works at temperatures above 90 °C while maintaining fluidity at lower temperatures. The fluid is converted into a stable gelled state through mixing [51]. The gelation mechanism diagram is shown in Figure 4. Machine learning integration will accelerate formulation optimization. It reduces R&D cycles by 50% through high-throughput screening [52].

Notably, mineral grinding technology (e.g., vibrating cup mill grinding) is critical for the preparation of fracturing-related nanomaterials—it enables the refinement of raw minerals into nanoscale particles with uniform size, providing a foundation for subsequent surface modification [53]. Under dry conditions (e.g., arid oilfields in the Permian Basin), nanoparticle-stabilized CO_2_ foam fracturing avoids water scarcity issues; under wet conditions (e.g., water-sensitive shale reservoirs), hydrophilic nano-Al_2_O_3_ inhibits clay swelling, reducing formation damage from water-based fluids. Mineral grinding technology can also be optimized to adjust proppant micro-morphology—for example, grinding quartz sand into micro-nano composites improves its compatibility with nanoparticles. Additionally, nanofracturing may influence seismic fault stability as nanoparticles can reduce fault surface friction (similar to the melt lubrication effect in earthquakes) [54], potentially mitigating induced seismicity risks.

## 5. Conclusions

Nanotechnology has emerged as a transformative force in fracturing. Nanotechnology addresses critical limitations of conventional techniques through material innovation. It enhances high-temperature viscosity retention by 3 times, increases proppant strength by 25%, and reduces water usage by 80% in waterless fracturing. With these improvements, this technological advancement enables efficient development of unconventional reservoirs.

The technology’s ability to solve interconnected challenges is notable. These challenges range from fluid stability to post-fracturing cleanup, highlighting the technology’s systemic value. Field tests provide concrete evidence of its benefits. They show 10–18% production increases, 85% conductivity recovery, and 70% lower flowback treatment costs. These results underscore its practical potential.

Despite hurdles in agglomeration, cost, and ecotoxicology, ongoing research is paving the way for industrial deployment. Research into biodegradable materials, intelligent systems, and waste-derived nanoparticles is making progress. Nanotechnology is poised to play a central role in the global energy transition. It enables low-carbon, efficient extraction of unconventional resources while minimizing environmental impact. Its continued advancement will be critical to meeting future energy demands sustainably.

## Data Availability

No new data were created or analyzed in this study.
